# Alcoholisme Et Criminalité

**Published:** 1889-05

**Authors:** 


### BOOK NOTICES AND REVIEWS.

Alcoholisme et Criminalité, traitement Médical de l'ivrognerie et de l'ivresse. Par le Dr. Gallavardin, de Lyon, en France. 1889.

In this brochure Dr. Gallavardin points out the well known evil effects of chronic alcoholism; to its baneful effects most of the crime and many of the current diseases are attributed.

